# Most Early‐Born Subplate Neurons Persist as Layer 6b Neurons in the Adult Mouse Neocortex

**DOI:** 10.1002/cne.70200

**Published:** 2026-09-28

**Authors:** Yusuke Sugita, Keiko Moriya‐Ito, Carina Hanashima, Chiaki Ohtaka‐Maruyama

**Affiliations:** ^1^ Developmental Neuroscience Project, Department of Basic Medical Sciences Tokyo Metropolitan Institute of Medical Science Tokyo Japan; ^2^ Graduate School of Advanced Science and Engineering Waseda University Tokyo Japan

**Keywords:** birthdate analysis, neocortex, postnatal development, subplate, tissue clearing

## Abstract

Subplate neurons (SpNs) are early‐born neurons in the mammalian neocortex and play a pivotal role in cortical development. It is widely believed that SpNs are predominantly lost due to postnatal cell death, with only a few remnants contributing to layer 6b (L6b) neurons in the adult neocortex, but the extent to which SpNs persist as L6b neurons remains largely unknown. To address this, we conducted a comprehensive birthdate analysis using multiple ethynyl deoxyuridine (EdU) injections over 24 h, thereby precisely tracking the survival and distribution of SpNs in the adult mouse neocortex. We further estimated the total number of SpNs and L6b neurons based on the total volumes of the subplate and L6b, quantified by employing 3D tissue clearing. Our findings reveal that a significant proportion of L6b neurons are remnants of SpNs and comprise diverse subtypes reminiscent of those in the postnatal period (SpNs: 319,588 ± 49,263, L6b neurons: 316,720 ± 11,268). This study provides direct evidence showing that most L6b neurons in the adult mouse neocortex originate as SpNs during the earliest stages of neurogenesis and persist throughout life.

## Graphical abstract

Comprehensive EdU birthdating and 3D volumetric analyses revealed that most early‐born subplate neurons (SpNs) persist into adulthood as layer 6b (L6b) neurons in the mouse neocortex. Green circles indicate SpNs/L6b neurons, and magenta labeling indicates EdU‐labeled early‐born neurons.

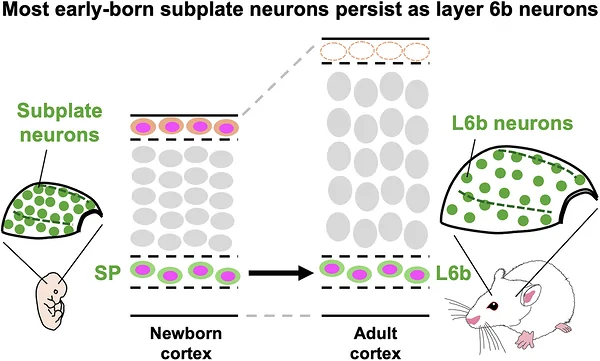

## 1 Introduction

The mammalian neocortex contains a distinct six‐layer structure, with layer 6b (L6b) constituting a thin cellular sheet located directly above the white matter (WM). L6b neurons project to multiple intracortical and extracortical regions, including layer 1, layer 5a, and the medial posterior thalamic nucleus (POm; Chang et al. 2024; Hoerder‐Suabedissen and Molnar 2013; Hoerder‐Suabedissen et al. 2018; Kim et al. 2025; Viswanathan et al. 2017; Zolnik et al. 2024). Emerging evidence highlights the unique physiological roles of L6b neurons in the adult brain, particularly in the regulation of sleep‐wake states and cognitive processing (Ben‐Simon et al. 2022; Clancy and Cauller 1999). Notably, a subset of deep‐layer neurons specifically express orexin receptor type 1 and type 2 mRNA (Tsuneoka and Funato 2024); and only L6b neurons respond to Orexin B across the neocortex, suggesting that they are involved in sleep state regulation (Bayer et al. 2004). Furthermore, entorhinal L6b neurons project to the hippocampus, contributing to spatial coding and memory in mice (Ben‐Simon et al. 2022). Therefore, despite their low density, L6b neurons are essential for integrating information in the adult neocortical circuitry.

However, the developmental origin of L6b neurons remains unclear. In the developing neocortex, the deepest layer of the cortical plate—corresponding to the adult L6b—is called the subplate (SP; Feldmeyer 2023; Kanold and Luhmann 2010; Molnar et al. 2020), which comprises neurons (SpNs) embedded within an abundant extracellular matrix. SpNs are produced during the earliest phase of neurogenesis, together with Cajal–Retzius (CR) cells, serving as fundamental components of the preplate (Hoerder‐Suabedissen and Molnar 2015; Kanold 2009; Luhmann et al. 2018; Ohtaka‐Maruyama 2020; Wang et al. 2010) and are crucial in forming the embryonic neocortex. They establish transient synaptic connections with migrating excitatory neurons to facilitate their multipolar‐to‐bipolar transition (Ohtaka‐Maruyama et al. 2018) and act as temporary targets for thalamocortical axons, guiding them toward layer 4 to establish mature thalamocortical circuits (Doyle et al. 2021; Hoerder‐Suabedissen and Molnar 2015; Meng et al. 2014; Molnar and Kwan 2024).

Although SpNs and L6b neurons occupy the same anatomical position, namely, the deepest part of the cortical plate, they are considered distinct due to their functional differences observed across developmental stages (deFazio et al. 1987; Marx et al. 2017; Rosen and Harry 1990; Woo et al. 1991). Conversely, some evidence suggests that a subset of SpNs survives as a uniform layer, referred to as layer 6b or layer 7 (Marx et al. 2017). During this process in the rodent neocortex, the observed decline in SpN density has been attributed primarily to cell death in the SP (Allendoerfer and Shatz 1994). Although early birthdating studies in rats suggested that a substantial fraction of SpNs persists into adulthood and contributes to L6b (Valverde et al. 1995) or explains the morphological similarities (Marx et al. 2017), these analyses relied on single‐pulse radiolabeling. Such methods capture only a narrow temporal window (∼4 h) and label a small subset of the population (deFazio et al. 1987), lacking the quantitative, whole‐cortex resolution required to resolve the issue regarding SpN persistence.

This uncertainty originates from three primary technical challenges (Feldmeyer 2023; Marx et al. 2017): (1) The molecular heterogeneity of SpNs and the lack of universal SpN‐specific markers hinder their comprehensive characterization; (2) the massive postnatal expansion of the neocortex, including the SP, makes it difficult to assess the precise structure and distribution of SpNs; and (3) the limitations of single‐pulse thymidine analog injections inhibit reliable labeling of the entire early‐born population. While some of the labeled neurons are present in L6b, unlabeled neurons may either represent other types of neurons or early‐born neurons that failed to take up the analog. Previous studies have suggested that the developmental decline in SpN density can be explained by the increase in total brain volume (Rosen and Harry 1990; Woo et al. 1991). However, these studies did not directly measure postnatal volume changes in SP and L6b, and thus, the actual change in SpN number may not match the calculated value, necessitating re‐evaluation of the assumption that the number of SpN decreases postnatally.

To overcome these limitations, we employed a saturated ethynyl deoxyuridine (EdU) labeling protocol (every 4 h between E10.5 and E11.5 and every 6 h between E11.5 and E12.5) and CUBIC‐based tissue clearing (Matsumoto et al. 2019) to visualize and measure the entire SP and L6b volumes. Using NeuN immunostaining on brain sections at two stages, P0 and 8 weeks, we quantified the number of neurons as NeuN‐positive cells and multiplied these counts by the L6b volumes to estimate the total number of SpN in the neocortex. The results reveal that most early‐born SpNs persist up to 8 weeks, contrary to previous assumptions. These findings support two conclusions: L6b neurons are survivors of early‐born SpNs, and in the developing mouse brain, there is no significant decline in early‐born SpNs, with many persisting in the adult L6b.

## 2 Materials and Methods

### 2.1 Animals

All experimental protocols were approved by the Animal Care and Use Committee of the Tokyo Metropolitan Institute of Medical Science (23‐008, 24‐005). Animals were provided food and water ad libitum and maintained on a 12‐h light/dark cycle. Lpar1‐EGFP males (Tg[Lpar1‐EGFP]GX193Gsat/Mmucd, RRID: MMRRC_030171‐UCD) were mated with wild‐type ICR female mice (LSC, Japan) for 12 h overnight with Lpar1‐EGFP males and checked for plugs; E0.5 was defined as the first midday after checking plugs. ICR females were selected for their higher pup yields and superior resilience to the stress of multiple thymidine analog injections, compared with B6 black females.

### 2.2 EdU Treatment and Tissue Collection

For the single‐injection groups, pregnant dams were injected with EdU once at E11.5 or E12.5 (15 mg/kg in sterile saline; Mase et al. 2021). For E11 whole labeling (E11wl), intraperitoneal EdU injection was performed once every 4 h (six times) from E10.5 to E11.5, and for E12 whole labeling (E12wl), intraperitoneal EdU injection was performed once every 6 h (four times) from E11.5 to E12.5. Wild‐type and Lpar1‐EGFP (male, with the Lpar1‐EGFP transgene located on the Y chromosome) pups were perfused with 4% paraformaldehyde (PFA) on E17.5, P0, P14, and 8 weeks (P56–60). Brains were dissected and fixed in 4% PFA/PBS overnight (24 h) at 4°C.

### 2.3 Antibodies

The following primary antibodies were used: anti‐CTGF (goat, Santa Cruz Biotechnology, sc‐14939, RRID: AB_638805), anti‐NeuN (mouse, Cell Signaling Technology, 94403, RRID: AB_2904530), anti‐NR4A2 (goat, R&D Systems, AF2156, RRID: AB_2153894), anti‐GFP (chicken, Abcam, ab13970, RRID: AB_300798), anti‐TBR1 (rabbit, Abcam, ab31940, RRID: AB_2200219), and anti‐Cleaved Caspase‐3 (Asp175) (rabbit, Cell Signaling Technology, 9661, AB_2341188). Secondary antibodies comprised anti‐mouse 405 (donkey, Jackson, 715‐475‐150, RRID: AB_2340839), anti‐goat 488 (donkey, Invitrogen, A‐11055, RRID: AB_2534102), anti‐chick 488 (donkey, Jackson, 703‐545‐155, RRID: AB_2340375), anti‐mouse Cy5 (donkey, Jackson, 715‐175‐150, RRID: AB_2340819), anti‐rabbit Cy5 (donkey, Jackson, 711‐175‐152, RRID: AB_2340607), anti‐goat 633 (donkey, Invitrogen, A21082, RRID: AB_141493), and anti‐rat 633 (goat, Invitrogen, A21094, RRID: AB_141553). All antibodies were used at a 1:500 dilution.

### 2.4 Immunohistochemical and EdU Staining

Fixed brains were cryoprotected in 15% sucrose/PBS overnight, followed by 30% sucrose/PBS overnight at 4°C. The tissues were sectioned coronally at 20 µm using a cryostat (CryoStar, NX50) for brains aged E17.5 and P0 and at 30 µm using a microtome (Thermo Fisher Scientific, HM440) for brains aged P4, P7, P10, P14, and 8 weeks.

Sections were soaked three times in PBS for 10 min and pre‐incubated with 0.3% Triton X‐100/PBS for 2 h, which was then incubated overnight at 4°C with primary antibodies diluted in PBS containing 0.5% skim milk. After washing three times with PBS, the sections were incubated with secondary antibodies. Then, sections were mounted with PermaFluor (Thermo Scientific) after 4’,6‐diamidino‐2‐phenylindole (DAPI) staining (5 µg/mL, Sigma‐Aldrich, D9542). EdU staining was performed using a Click‐iT EdU Alexa Fluor 555 Imaging Kit (Invitrogen by Thermo Fisher Scientific, C10338). Z‐stack images of the immunostained sections were acquired at 4‐µm intervals across the full thickness of each section using a confocal microscope (LSM710, Zeiss). The resulting image stacks were processed into orthogonal projection images for visualization.

### 2.5 Definition of the SP/L6b Region

In later embryonic and early postnatal stages, the SP layer was defined as a 50‐µm‐thick band immediately adjacent to the WM boundary, as described in a previous study (Hoerder‐Suabedissen and Molnar 2013). The 50‐µm definition was also applied to the adult L6b, referring to the expression pattern of established SpN/L6bN markers (Figure 1A).

**FIGURE 1:**
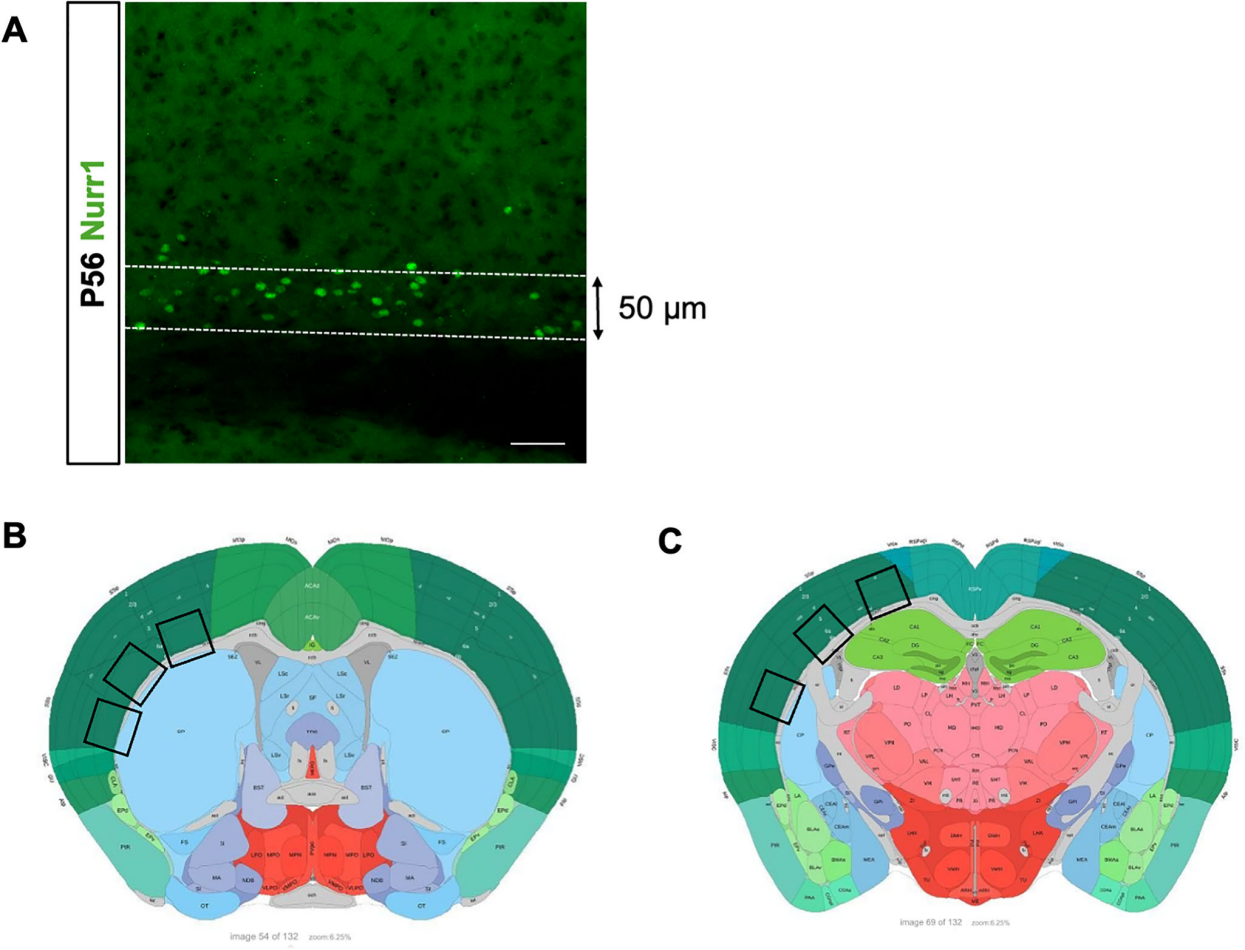
Window view of images used for quantification and definition of L6b. (A) Definition of the SP layer/L6b. The boundary between the SP layer/L6b and the neocortex was defined as 50 µm above the white matter. (B, C) Serial coronal sections from the mouse brain atlas, namely, Allen brain atlas No. 54 (B) and No. 69 (C). Black squares indicate the imaging area used for quantification (a total of six fields).

### 2.6 Cell Counting and SP/L6b Density Calculation

For cell counting, two coronal sections per brain were selected from all adult samples based on the Allen Brain Atlas (https://brain‐map.org/atlases/anatomy, section Nos. 54 and 69). Three images were captured from predefined neocortical regions in each section (Figure 1B,C). These regions were selected based on consistent anatomical landmarks located along the rostrocaudal axis, and identical sampling positions were used across all samples to ensure comparability. In total, six images per brain were analyzed. Within each image, the SP/L6b region of interest (ROI) was manually defined in Fiji ImageJ (RRID: SCR_002285) as a rectangular band extending 50 µm from the WM boundary toward the pial surface. NeuN+, EdU+, and SP marker‐positive cells within this ROI were manually counted using the ImageJ Cell Counter plugin. To avoid double‐counting, serial z‐stack images were inspected during counting. Because CTGF is predominantly localized in the cytosol and does not clearly delineate cell boundaries, NeuN staining was used to accurately define individual neurons and improve the reliability of cell counting. SP/L6b density was defined as the number of neurons within the SP/L6b region divided by its area, and six measurements from six ROIs were averaged and treated as a single data point for each brain. The number of neurons was determined by identifying NeuN+ cells (representing all SP/L6b neurons) and EdU+ cells (representing birthdated SP/L6b neurons) within the ROI. The SP/L6b area was measured using the ImageJ Rectangle tool, according to the defined SP/L6b boundaries.

### 2.7 Tissue Clearing and Whole‐Brain Imaging

Newborn and adult whole brains were cleared using the CUBIC protocol (Matsumoto et al. 2019; Susaki et al. 2015). Lpar1‐EGFP mice (P0 and 8 weeks) were fixed by perfusing with 4% PFA for 3 days at 4°C. The fixed brains were washed three times in PBS for 6 h at room temperature and incubated in 4 mL 0.5 × CUBIC‐L (1:1 N‐butyldiethanolamine and TritonX‐100 in water) in PBS overnight. The solution was then replaced with 2 mL 1 × CUBIC‐L and delipidated three times at 37°C with gentle shaking overnight. The samples were washed three times in PBS for 6 h at room temperature. The samples were incubated in 0.5 × CUBIC‐R (Antypirine, N‐methylnicotinamide, and N‐butyldiethanolamine in water) for 6 h. Then, they were replaced twice with 1 × CUBIC‐R at 37°C with gentle shaking overnight. Cleared whole‐brain tissues were imaged by light‐sheet fluorescence microscopy (LSFM; Olympus, MVX10‐LSFM); the raw images were acquired horizontally at z‐axis intervals of 4 µm in the P0 samples and 9 µm in the 8‐week samples.

## 3 3D Reconstruction

For 3D reconstruction, the GFP‐positive neocortical SP/L6b region was manually identified in each light‐sheet image, and the corresponding area was measured in ImageJ for each horizontal optical plane. The total SP/L6b volume was then estimated by summing the measured areas across the z‐axis and multiplying by the z‐step interval (4 µm at P0 and 9 µm at 8 weeks).

$$V=\sum \left(\frac{SP\;layer\;area}{{magnification}^{2}}\right)\times z-axis\;interval$$

### 3.1 Estimation of the Total SpNs/L6b Neuron Populations

To estimate the total number of SP/L6b neurons, we first calculated the volumetric NeuN+ cell density from the section‐based counts by dividing the areal density (cells/mm^2^) by the physical section thickness (20 µm at P0 and 30 µm at 8 weeks). The total number of SP/L6b neurons was then obtained by multiplying this volumetric density by the reconstructed SP/L6b volume.

### 3.2 Counting EdU+ L6b Neurons in the Entire Neocortex to Validate the Estimation

To cover the entire neocortex, each hemisphere was coronally sectioned into approximately 200 serial sections with thicknesses of 30 µm, and all sections were stained with DAPI and EdU. EdU+ cells in the SP/L6b were counted in all acquired images using ImageJ, following the same procedure described for cell counting. The density of EdU+ L6b neurons was calculated along the anteroposterior axis. The total number of EdU+ L6b neurons was obtained by summing the counts across all sections in each hemisphere.

### 3.3 Estimation of the CR Cells/L1 Neuron Populations

The marginal zone (MZ)/layer 1 (L1) region was defined based on its low signal intensity, compared with other cortical regions in horizontal optical sections of CUBIC‐cleared brains, as well as its low NeuN+ cell density in coronal immunostained sections. As with the estimation of SpN/L6b neurons, 3D reconstruction was performed by combining MZ/L1 area measurements obtained from horizontal sections with the CR cell/L1 neuron density calculated from NeuN+ cells within manually defined ROIs in coronal sections.

### 3.4 Statistical Analysis

Statistical analyses were performed using Microsoft Excel. All data were expressed as the mean ± S.D., and Welch's *t*‐tests (unpaired two‐tailed) were used to compare the means between two groups.

## 4 Results

### 4.1 EdU Administration Over 24 H Sufficiently Captures Early‐Born SpNs

To analyze the distribution of SpNs across multiple stages, we defined SpNs as early‐born neurons in the neocortical neurogenesis period (E10.5 to E12.5) located at the bottom edge of the cortical plate in mice (Hoerder‐Suabedissen and Molnar 2013). To comprehensively label SpNs generated during this period, we employed multiple injections of EdU, considering that the efficient labeling window of a single injection of thymidine analog is 2 to 6 h (deFazio et al. 1987). To avoid EdU overdose, we divided the SP neurogenesis period into two phases: E10.5–E11.5 and E11.5–E12.5. Six EdU injections were administered at 4‐h intervals for E11wl. For E12wl, four EdU injections were administered at 6‐h intervals to improve postnatal survival. For E11wl (Figure 2A), 60% of pups survived and grew to adulthood (*n* = 6 pregnant mice). For E12wl, almost all pups died due to stillbirth or parental abandonment (*n* = 12 pregnant mice). To circumvent this, we reduced the EdU injections to four times at 6‐h intervals, which allowed the pups to survive postnatally (Figure 2D). To assess the efficiency of this protocol in labeling SpNs, we compared it with a single thymidine analog injection, as employed in previous studies. In P0 mice, the EdU+ neurons in the neocortex in mice injected with EdU at E11.5 were restricted to the SP and the MZ, as expected (Figure 2B); however, the density of EdU+ neurons was approximately six times higher in the SP of E11wl than in that of the E11.5 single‐injection group (Figure 2C). Similarly, approximately five times higher EdU+ neuron labeling was observed in E12wl than in the E12.5 single‐injection group (Figure 2E,F). These results demonstrate that employing multiple EdU injections at 4‐ or 6‐h intervals promotes complete labeling of the early‐born neurons that were missed by a single injection. Next, we examined whether EdU+ neurons in the MZ were CR cells because CR cells are one of the earliest‐born neurons in the cerebral cortex and, along with SpNs, are expected to decline during postnatal development. We tracked the localization of EdU+ SpNs and CR cells during embryonic and postnatal stages. At E17.5 and P0, both EdU+ SpNs and CR cells were observed in their respective locations; however, at P14, almost no EdU+ cells were detected in the MZ. In contrast, in L6b, many EdU+ neurons persisted, and a small fraction of deep‐layer neurons were labeled by EdU at P14 (Figure 2G). This result suggests that EdU+ early‐born neurons persisted in SP but were limited in the MZ at P14. Thus, although SpNs and CR cells are both early‐born neurons, their postnatal reduction occurs in a significantly distinct manner.

**FIGURE 2:**
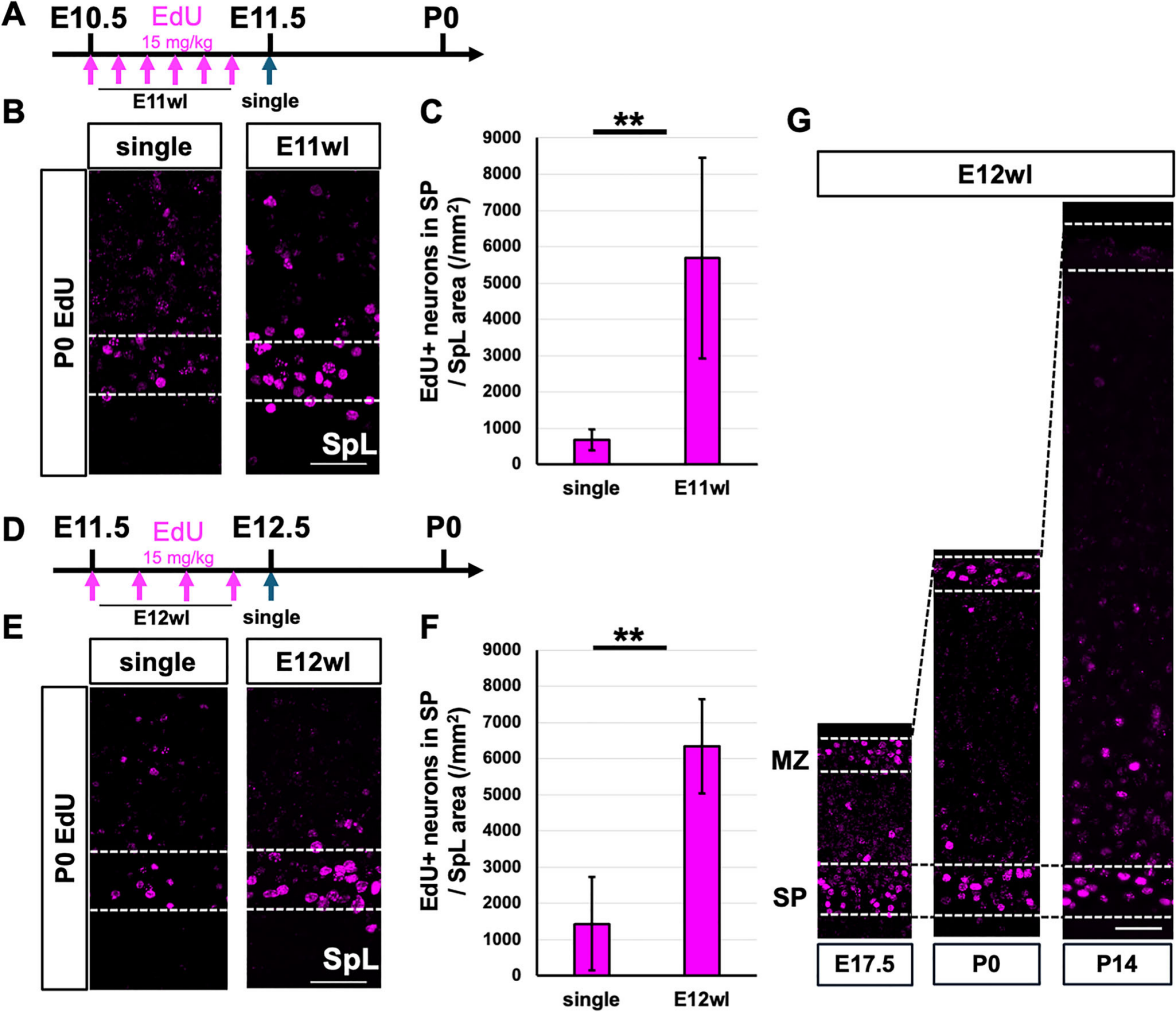
SpNs were efficiently labeled using the whole labeling method. (A) Schematic timeline of the whole labeling process with EdU. For E11 whole labeling (E11wl), EdU (15 mg/kg) was injected once every 4 h (six times) from E10.5 to E11.5. In the single‐injection group, EdU (15 mg/kg) was injected once at E11.5. Fixed samples were obtained at P0. (B) Distribution of EdU+ cells in the somatosensory cortex at P0 in the single‐injection and E11wl groups. Scale bars indicate 50 µm. (C) The density of EdU+ neurons in the subplate layer (SpL) area in the single‐injection (*n* = 6 fields of view, 3 animals) and E11wl (*n* = 6 fields of view, 3 animals) groups at P0. ^∗∗^ *p* < 0.01; Welch's *t*‐test (unpaired two‐tailed). (D) The schematic timetable of the whole labeling by EdU. For E12 whole labeling (E12wl), EdU (15 mg/kg) was injected once every 6 h (four times) from E11.5 to E12.5. In the single‐injection group, EdU (15 mg/kg) was injected once at E12.5. Fixed samples were obtained at P0. (E) Distribution of EdU+ cells in the somatosensory cortex at P0 in the single‐injection and E12wl groups. Scale bars indicate 50 µm. (F) The density of EdU+ neurons in the SpL area in the single‐injection (*n* = 6 fields of view, 3 animals) and E12wl (*n* = 6 fields of view, 3 animals) groups at P0. ^∗∗^ *p* < 0.01; Welch's *t*‐test (unpaired two‐tailed). (G) Distribution of E12wl EdU+ cells at E17.5, P0, or P14. The upper line indicates the MZ, and the bottom line indicates the SP layer. Scale bars indicate 50 µm.

### 4.2 Most L6b Neurons Are Generated Between E10.5 and E12.5

The comprehensive labeling method (E11wl and E12wl) enabled us to assess the number of embryonic SP neurons that survived into adulthood as L6b neurons in the neocortex. As a result, E11wl and E12wl EdU+ cells were specifically located in the L6b in the entire neocortex at 8 weeks (Figures 3 and 4). We then analyzed the extent of E12wl neurons throughout the entire neocortex, measuring the depth of the EdU+ neurons, which were located in a few coronal sections (Figure 5A). The thickness of the EdU+ regions was approximately 70 µm in the anterior, approximately 50 µm in the somatosensory area, and approximately 40 µm in the posterior, and the average depth across the entire EdU+ region was 50.6 µm (Figure 5B). Consistent with the notion that the SP thickness in the somatosensory cortex is 50 µm in the first postnatal week (Hoerder‐Suabedissen and Molnar 2013), we identified EdU+ neurons continuously distributed in the adult L6b, within 50 µm of the WM. These observations indicate that SP/L6b do not expand vertically during the postnatal period.

**FIGURE 3:**
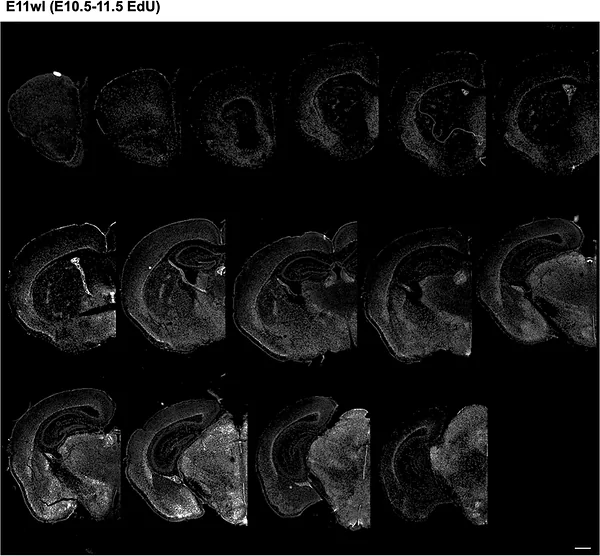
Serial coronal sections of an E11wl mouse brain. EdU‐positive cells represent neurons born within the E11 labeling window. Scale bars indicate 500 µm.

**FIGURE 4:**
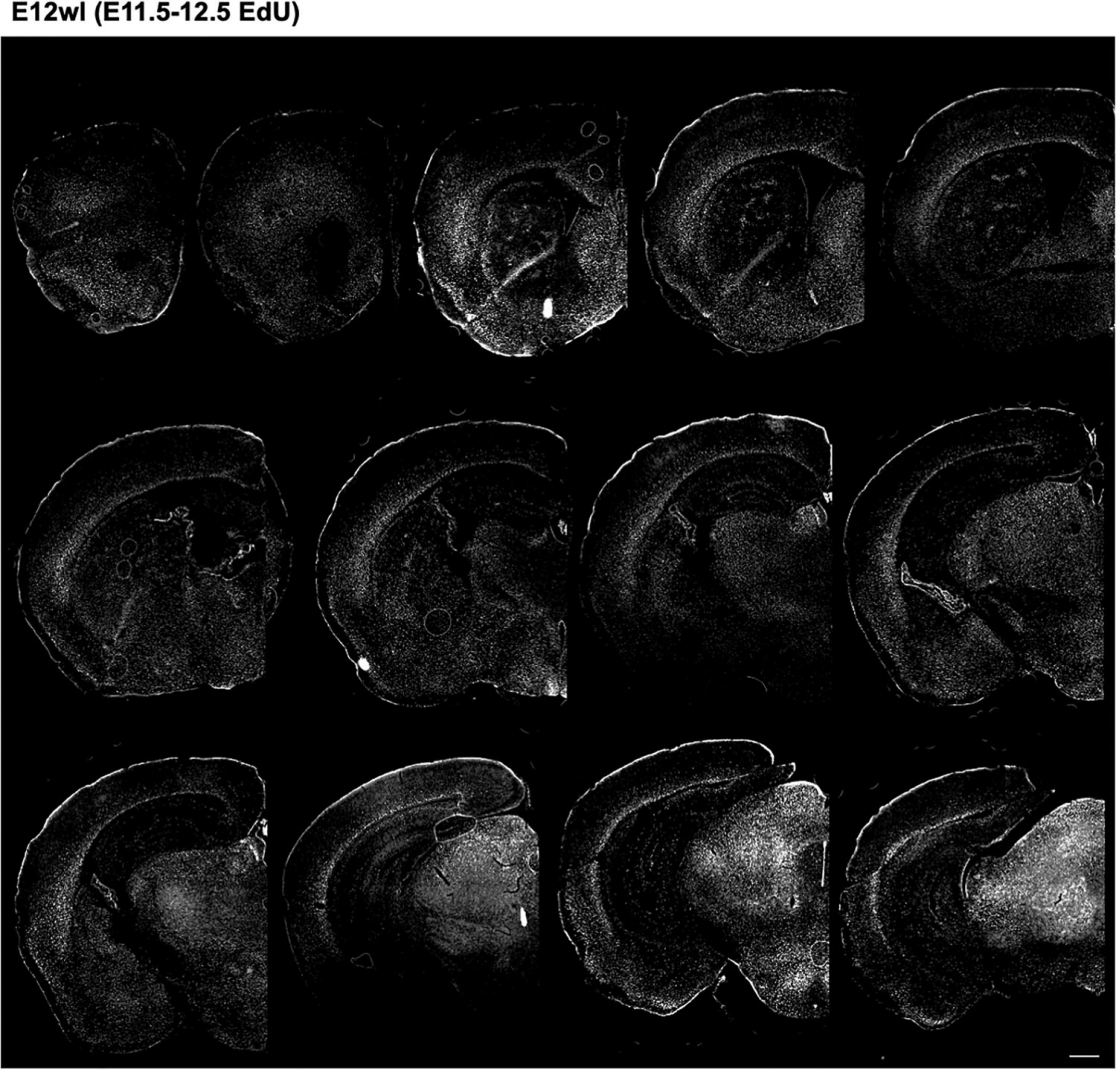
Serial coronal sections of an E12wl mouse brain. EdU‐positive cells represent neurons born within the E12 labeling window. Scale bars indicate 500 µm.

**FIGURE 5:**
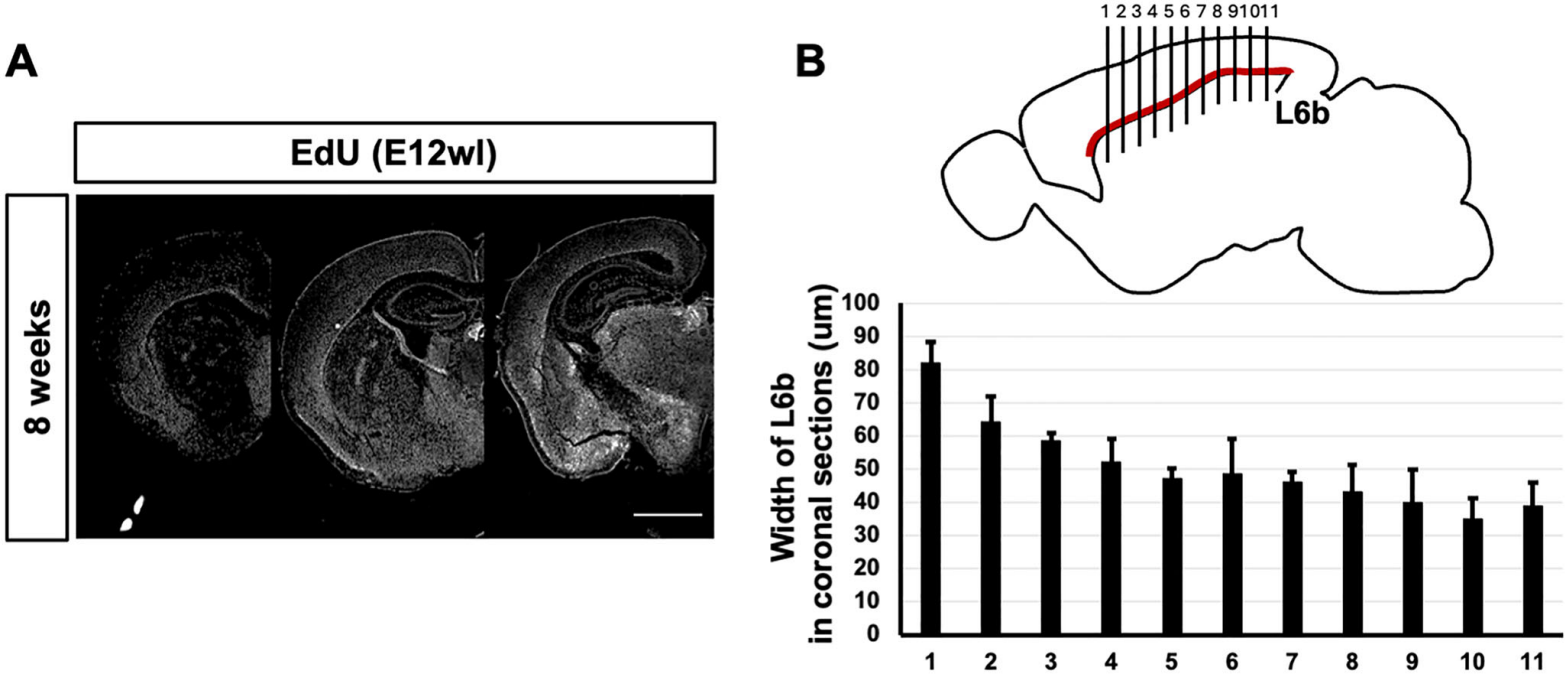
Distribution of EdU+ cells across coronal sections and the thickness of L6b neuron regions. (A) Series of confocal micrographs from coronal sections for E12wl EdU+ cells at 8 weeks, which were rostral, middle, or caudal. (B) The width of L6b at 8 weeks in the coronal section (µm). The horizontal axis indicates the position along the rostrocaudal axis, measured as the distance from the junction between the olfactory bulb and the neocortex (µm). The average width of L6b across all sections was 50.60 µm.

We also analyzed the percentage of EdU+ neurons in L6b neurons at 8 weeks. EdU+ early‐born neurons remained in the adult L6b, and the percentage of the EdU+ population in the E11wl group was 34.64% ± 13.23% (EdU+/NeuN+; *n* = 4, Figure 6A,C). These results indicate that the SpNs generated through E10.5–E11.5 represent approximately one‐third of the adult L6b population. We further quantified the E12wl EdU+ neurons, which occupied 64.01% ± 6.65% of the L6b neurons (EdU+/NeuN+; *n* = 4, Figure 6B,C). Collectively, these results indicate that more than 90% of adult L6b neurons were generated between E10.5 and E12.5 (Figure 6D). In contrast, few EdU+/NeuN+ cells were observed in the WM, indicating that these early‐born neurons were largely confined to L6b in the adult mouse neocortex. Thus, embryonic SpNs generated during this period persist as L6b neurons and are distributed throughout the L6b layer of the adult neocortex.

**FIGURE 6:**
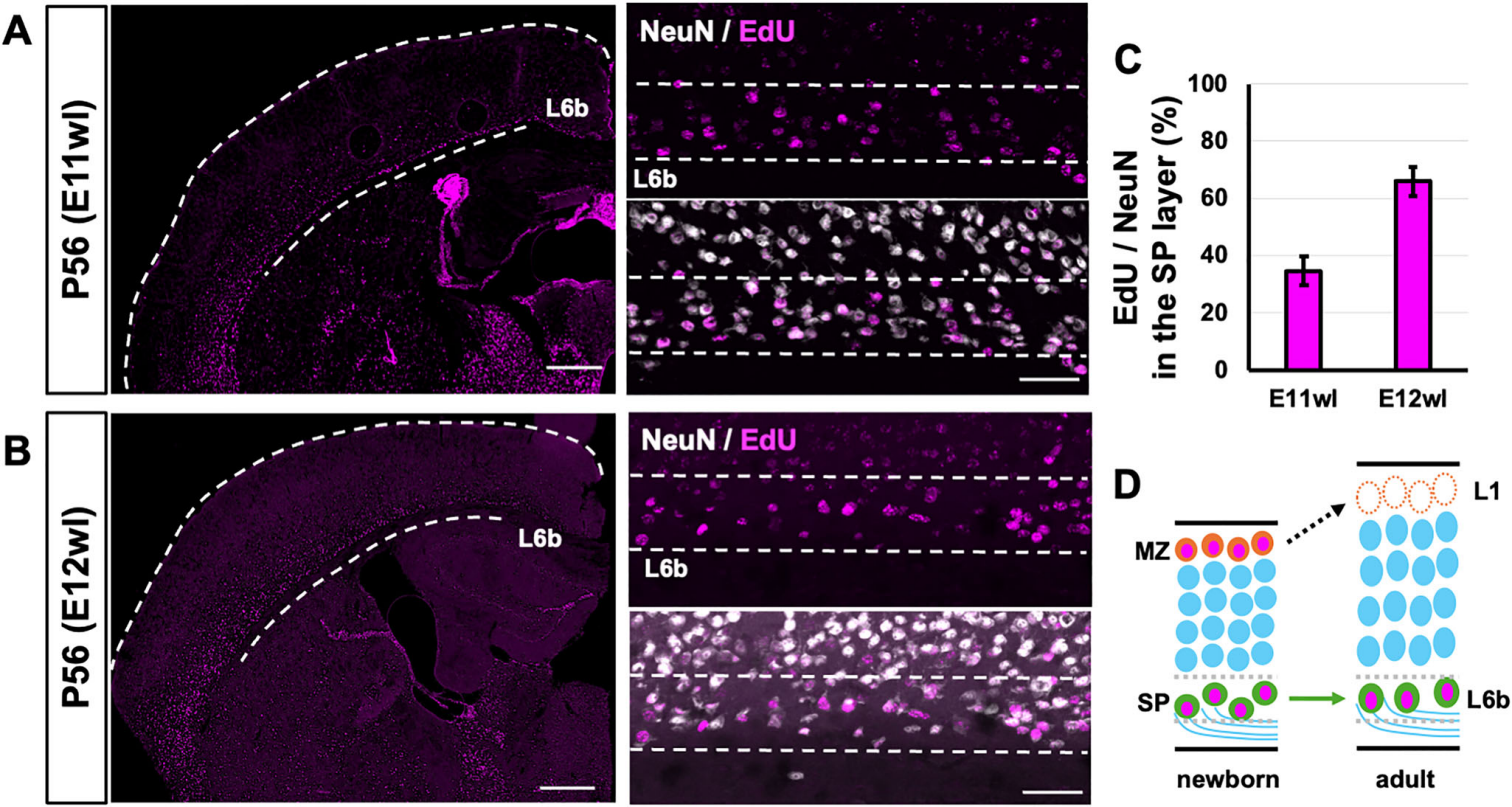
Early‐born neurons persist as L6b neurons in the adult cortex. (A) Confocal micrographs of E11wl (E10.5–E11.5) EdU+ cells at 8 weeks. The white dashed line indicates the lower boundary of L6b in the adult neocortex. Scale bars indicate 500 and 50 µm. (B) Confocal micrographs of E12wl (E11.5–E12.5) EdU+ cells at 8 weeks. The white dashed line indicates the lower boundary of L6b in the adult neocortex. Scale bars indicate 500 and 50 µm. (C) Proportion of EdU+ neurons (EdU+/NeuN+) among the total L6b neurons at 8 weeks. Data are shown for E11wl at 8 weeks (*n* = 4 brains, *n* = 24 images) and E12wl at 8 weeks (*n* = 4 brains, *n* = 24 images). (D) Summary of EdU+ SpN and L6b neurons by whole labeling in postnatal development. EdU+ neurons (magenta) are located at the SP and MZ in the newborn, and no EdU+ neurons exist at the MZ, but EdU+ neurons are observed in the adult L6b.

### 4.3 L6b Neurons Have Distinct Molecular Characteristics From SpNs

Considering the molecular diversity of SpNs during the early postnatal period (Hoerder‐Suabedissen and Molnar 2013), we investigated whether EdU+ early‐born neurons express SpN markers in the adult neocortex and whether a molecular signature between the E11wl and E12wl birthdates exists. We immunostained CTGF and NR4A2 as established SpN markers, and TBR1 as an L6 marker for E11wl and E12wl L6b (Figure 7A).

**FIGURE 7:**
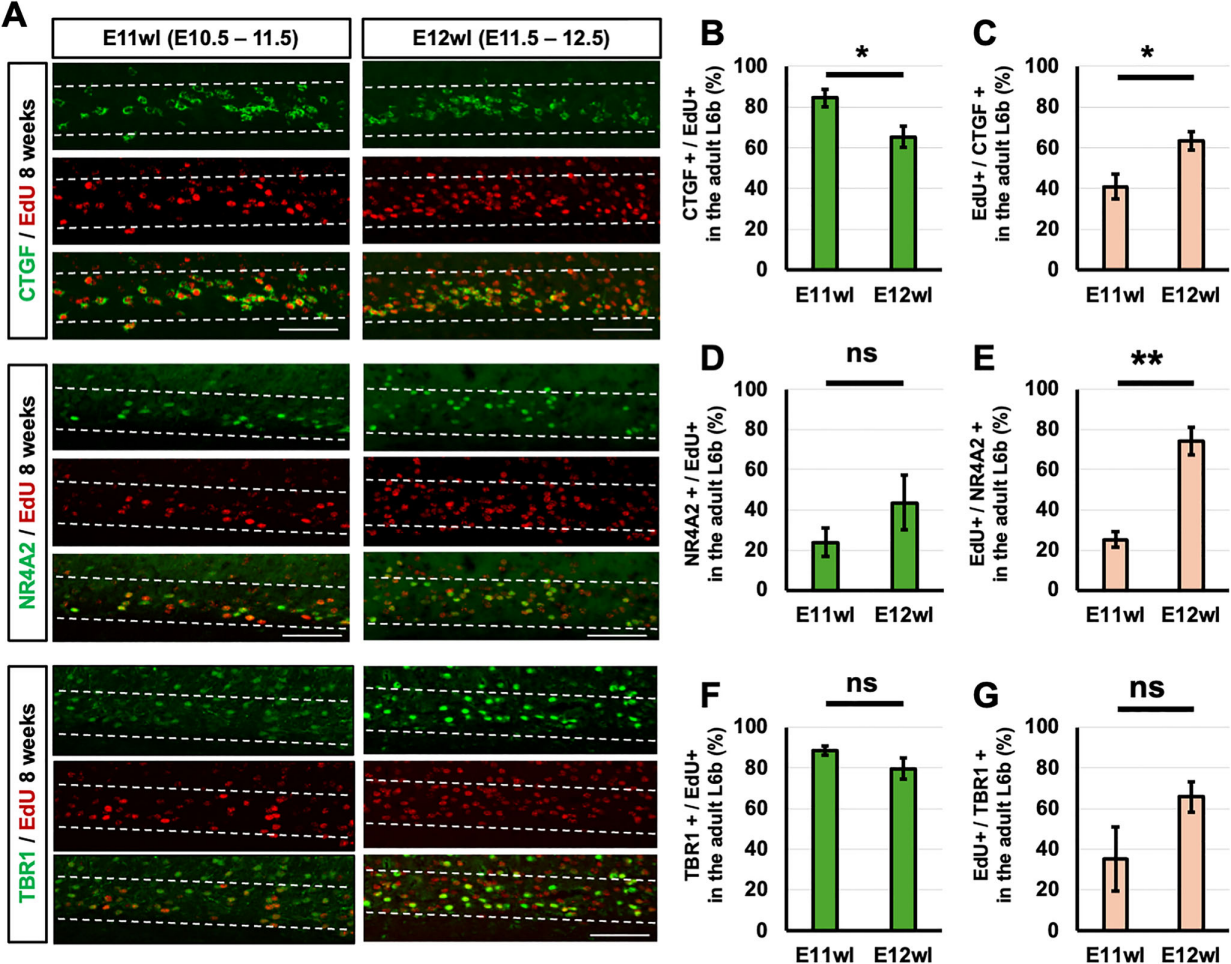
SpN marker+ neurons labeled with EdU persist in the adult L6b. (A) Immunohistochemical images showing CTGF (top), NR4A2 (middle), and TBR1 (bottom) expression in mice subjected to E11wl (left) or E12wl (right) and analyzed at 8 weeks of age. Scale bar = 50 µm (applies to all panels). (B, D, F) Bar graphs (mean ± SD) showing the percentage of EdU+ neurons that co‐expressed each marker: CTGF (B), NR4A2 (D), or TBR1 (F) at 8 weeks (*n* = 4 brains, *n* = 24 images). ^∗^ *p* < 0.05, ^∗∗^ *p* < 0.01, not significant (ns); Welch's *t*‐test (unpaired two‐tailed). (C, E, G) Bar graphs (mean ± SD) showing the percentage of each marker+: CTGF (C), NR4A2 (E), or TBR1 (G) neurons that colocalize with EdU+ nuclei for injections as E11wl and E12wl at 8 weeks (*n* = 4 brains, *n* = 24 images).^∗^ *p* < 0.05, ^∗∗^ *p* < 0.01, not significant (ns); Welch's *t*‐test (unpaired two‐tailed).

For SpN markers, CTGF expression was significantly higher in the E11wl population than in the E12wl population (CTGF+ & EdU+/EdU+: 84.54% ± 4.26% in E11wl and 65.33% ± 5.18% in E12wl, Figures 7B and 8). Assessing the birthdate of CTGF expressing cells within L6b neurons revealed a significantly higher E12wl population than E11wl population (CTGF+ & EdU+/CTGF+: 40.94% ± 6.17% in E11wl and 63.35% ± 4.35% in E12wl, Figures 7C and 8). NR4A2 was also expressed at a similar percentage; however, the percentage was relatively low compared with that of CTGF (NR4A2+ & EdU+/EdU+: 23.95% ± 6.90% in E11wl and 43.66% ± 13.46% in E12wl, Figure 7D). Notably, the NR4A2+ cell percentage significantly differed between the two birthdates, where NR4A2 represented a significantly higher number in E12wl cells (NR4A2+ & EdU+/NR4A2+: 25.18% ± 3.93% in E11wl and 73.93% ± 6.89% in E12wl, Figure 7E). For both SpN markers, the total percentage in E11wl and E12wl was approximately 100%. Moreover, according to our observations (Figure 6C), the difference between the expression rates was reasonable considering the number of L6b neurons. However, the percentage of NR4A2+ in E12wl was two‐fold higher than that in E11wl, indicating that SpNs generated at a later phase of SpN‐genesis are more likely to express NR4A2.

**FIGURE 8:**
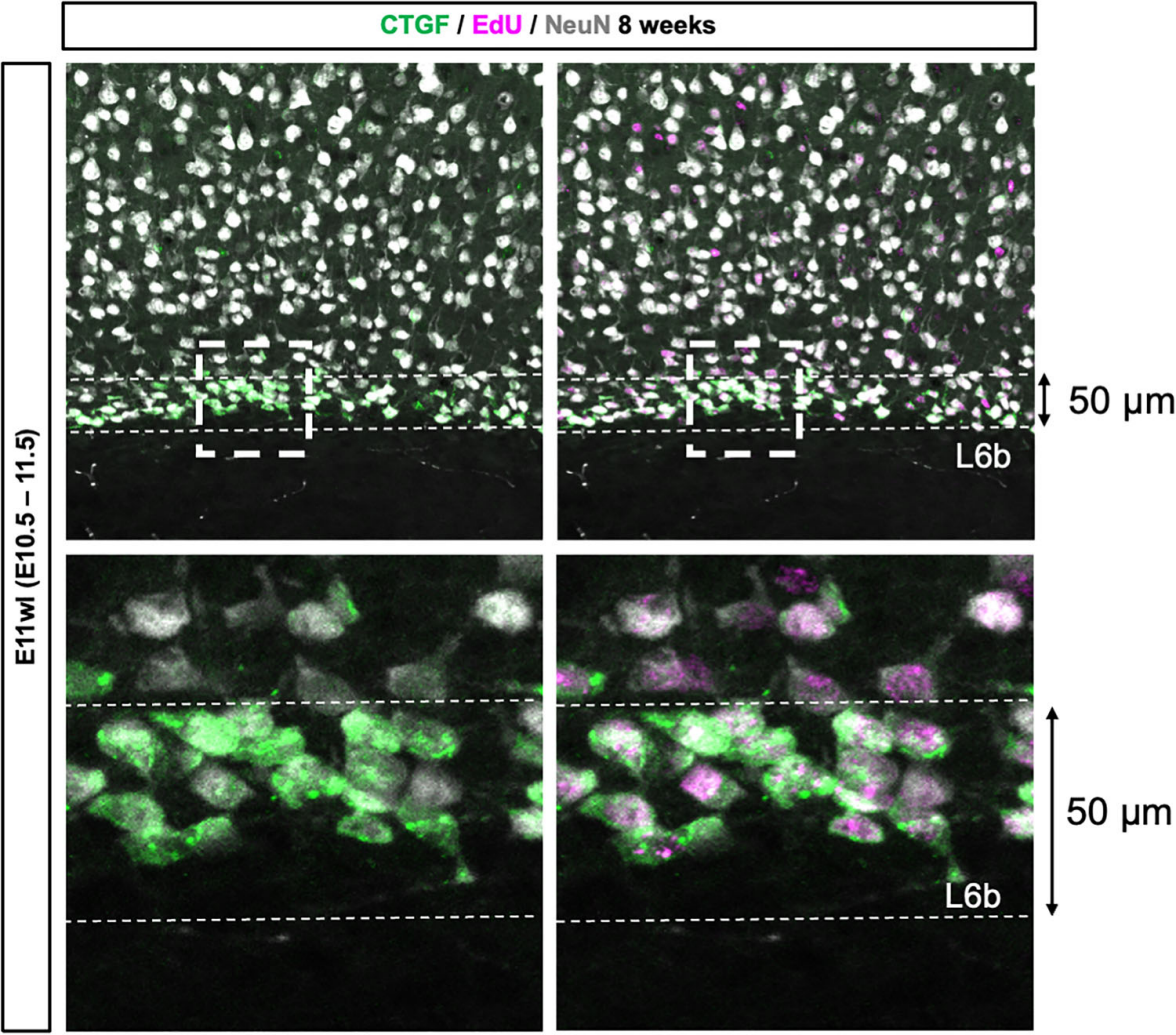
EdU+ L6b neurons cell counting with CTGF and NeuN immunostaining. Immunohistochemical images showing CTGF (green) and NeuN (gray) (left panel), and CTGF (green), EdU (magenta), and NeuN (gray) (right panel) expression in mice subjected to E11 whole labeling (E11 WL; left) and analyzed at 8 weeks of age.

We next examined L6 marker TBR1, which was highly expressed in both populations (TBR1+ & EdU+/EdU+: 88.62% ± 2.21% in E11wl and 79.60% ± 5.13% in E12wl, Figure 7F). Although the percentage of TBR1‐expressing L6b neurons did not significantly differ between the two birthdates, the E12wl population represented a higher proportion of TBR1+ cells than the E11wl population (EdU+ & TBR1+/TBR1+: 35.22 ± 15.63% in E11wl and 65.86 ± 7.39% in E12wl, Figure 7G). Therefore, similar to NR4A2, SpNs from the later phase of neurogenesis are more likely to express TBR1. Together, these results indicate that the surviving SpNs in L6b share common molecular identities with newborn SpNs. The surviving SpNs expressed both SP markers and, to some extent, L6 markers, suggesting that these early‐born SpNs retained in L6b contribute to the adult neuronal circuit through the expression of these markers.

### 4.4 Programmed Cell Death (PCD) Is Not the Main Cause of the Decline in SpN Density

In the adult neocortex, L6b neurons mainly consist of remnant SpNs. However, anatomical studies have shown that the density of SpNs/L6b neurons decreases during postnatal development (Hoerder‐Suabedissen and Molnar 2013; Price et al. 1997; Woo et al. 1991). To confirm this, we examined the developmental changes in neuron density using NeuN as an immunostaining neuronal marker and found a reduction in the density of SP/L6b neurons during the first postnatal week (Figure 9). It has been hypothesized that this decrease results from PCD during postnatal development (Luhmann et al. 2018; Wong and Marin 2019). To investigate whether PCD occurs within the SP during this period, we performed immunostaining using Caspase‐3 and assessed PCD at multiple postnatal stages. We first examined P4 (Figure 10A) brains due to the large decrease in cell density at this stage. Caspase‐3+ cells were observed throughout the neocortex but were not specifically concentrated in the SP. Immunohistochemistry at P4, P7, P10, and P14 (Figure 10B) revealed Caspase‐3+ cells in the neocortex, including the SP, but the overall number of Caspase‐3+ cells in the neocortex was very low (1–2 cells per field of view). Moreover, comparing the number of Caspase‐3+ cells between the SP and L6a revealed no significant differences (data not shown). We further assessed whether these Caspase‐3+ cells within the SP include glial cells, in addition to neurons, using NeuN for immunostaining (Figure 10C). Approximately 60% of the Caspase‐3+ cells in the SP co‐expressed NeuN, confirming their neuronal identity. However, approximately 40% of the Caspase‐3+ cells did not express NeuN. These cells may represent glial cells, or NeuN expression may be reduced or altered under apoptotic or specific cellular conditions, leading to their under‐detection. These findings suggest that PCD plays a limited role in the reduction of SpN populations during postnatal development. Therefore, the observed decrease in SpN density cannot be fully explained by cell death alone, raising the possibility that other factors, such as postnatal expansion of the neocortex, contribute to this apparent reduction.

**FIGURE 9:**
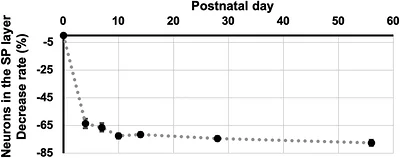
Decrease rate of the neuronal density in the SP. Decrease rate of the neuronal (NeuN+) density in the SP during postnatal development: (the density in each stage—the density at P0)/(the density at P0) × 100 (*n* = 3 brains, *n* = 18 images).

**FIGURE 10:**
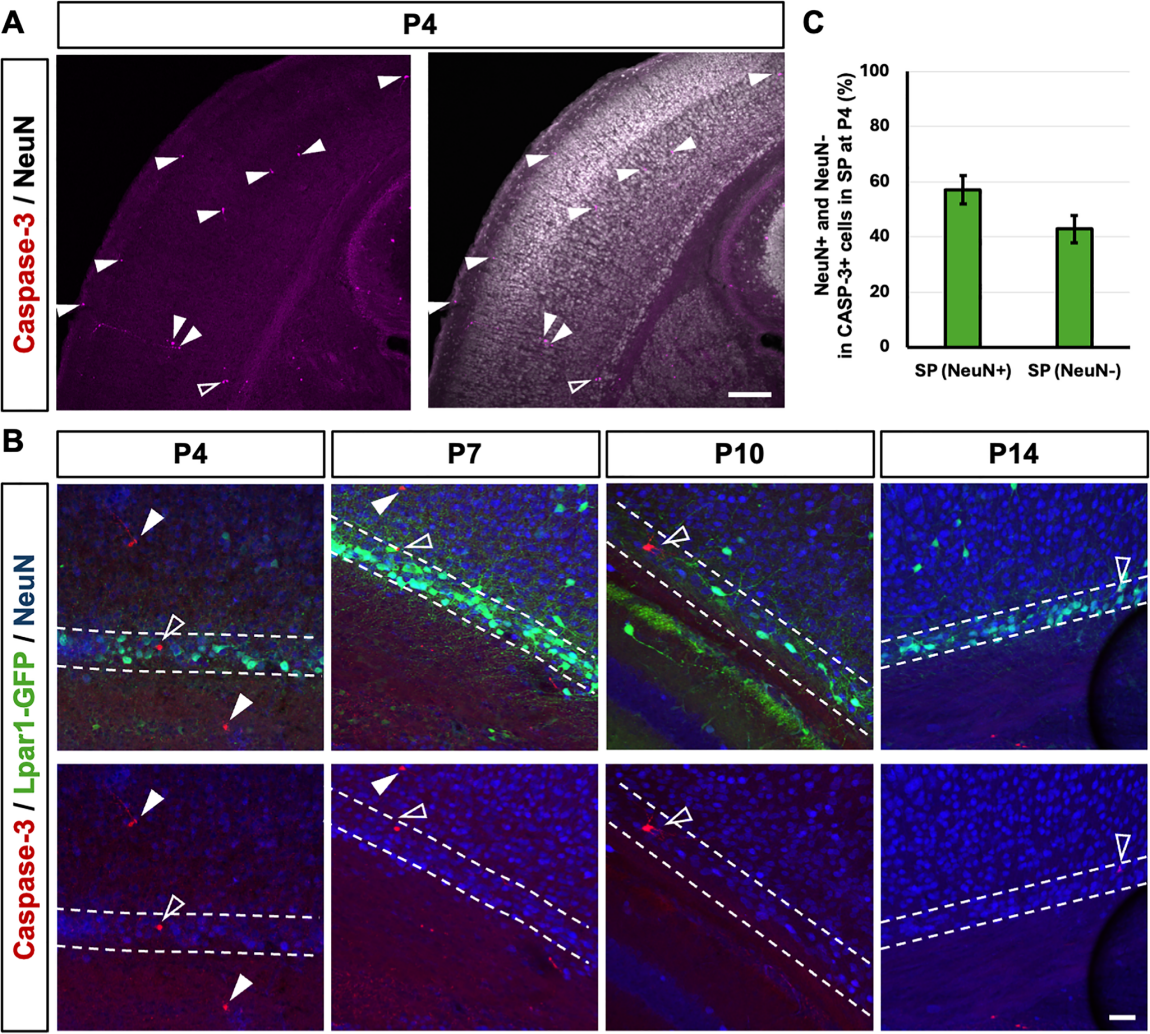
Few apoptotic neurons detected by Caspase‐3 were observed in the SP during postnatal development. (A) Immunohistochemical images of Caspase‐3 (magenta) and NeuN (gray) at P4. White closed arrowheads show Caspase‐3+ cells in the cortex, and white open arrowheads show Caspase‐3+ in the SP. Scale bar = 150 µm. (B) Immunohistochemical images of Caspase‐3 (red), Lpar1‐EGFP (green), and NeuN (blue) at P4, 7, 10, and 14. White closed arrowheads show Caspase‐3‐positive cells in the cortex, and white open arrowheads show Caspase‐3+ in the SP. White dashed lines denote the SP boundaries. Scale bar = 50 µm. (C) The percentage of NeuN+ or NeuN‐ among Caspase‐3 positive cells in the SP at P4. Caspase‐3 positive cells were counted from 8 slices in each brain and averaged as one sample.

### 4.5 Whole‐Brain Imaging Reveals Minimal Elimination of SpNs

The observed decrease in SpN density may be influenced by technical limitations associated with counting the total number using a series of brain sections (Figure 9). Note that the images from different stages were compared using the same field of view, without accounting for the volumetric expansion of the neocortex. We demonstrated that both the SP and L6b area expand tangentially during the first postnatal week (Figure 11A,B), particularly along the anteroposterior axis of the neocortex (Figure 11C,D). Thus, we hypothesized that neocortical expansion during this period accounts for the decrease in SpN density.

**FIGURE 11:**
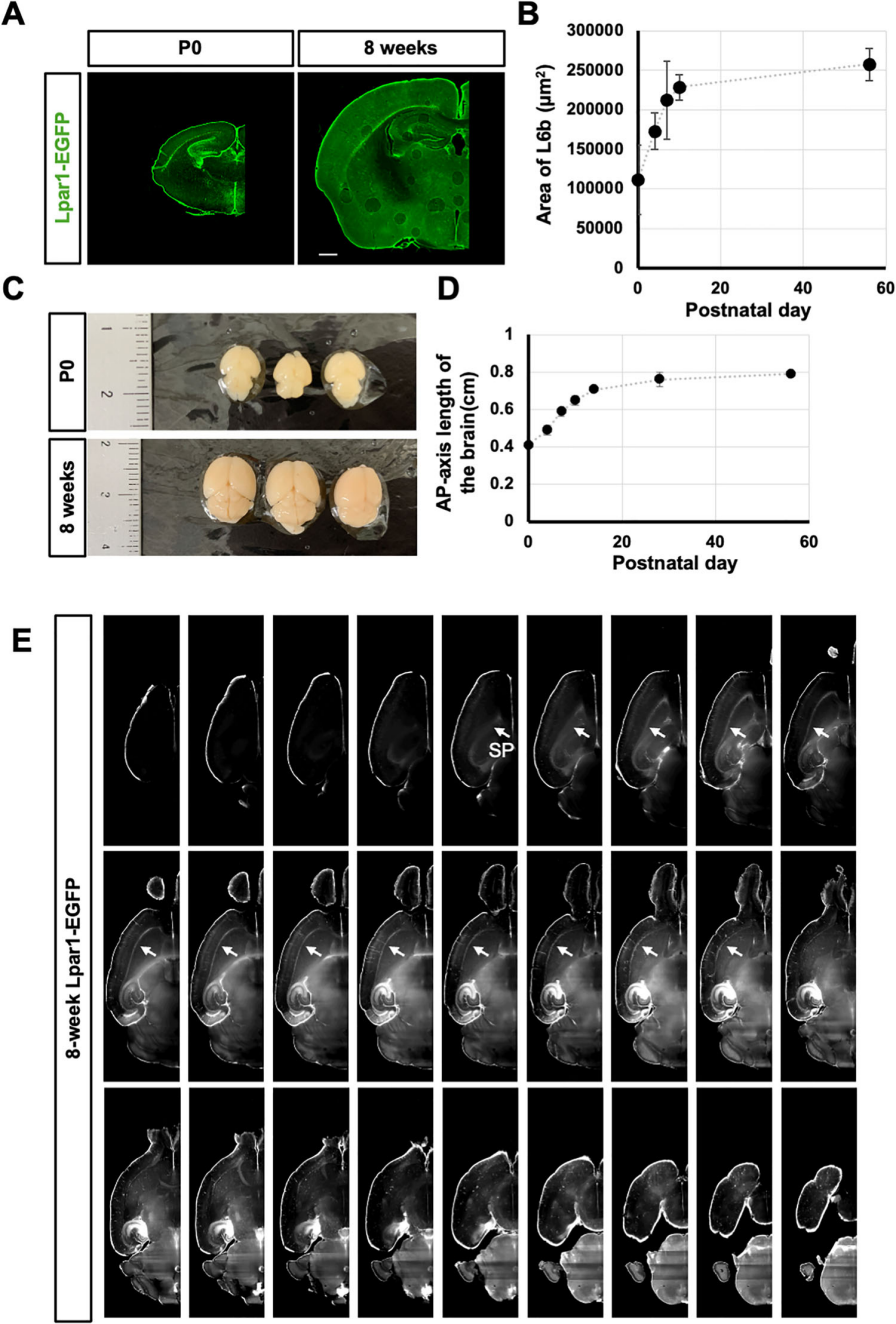
Postnatal volume expansion of the mouse cortex. (A) Immunohistochemical images of the coronal sections of Lpar1‐EGFP mouse brain at P0 (left) and 8 weeks (right). (B) Area of SP/L6b is defined by Lpar1‐EGFP expression (µm^2^; *n* = 3). (C) Photographs of 4% PFA‐fixed mouse brain at P0 (top) and 8 weeks (bottom). (D) Anteroposterior (AP) length of the brain excluding the cerebellum (cm; *n* = 3). (E) Series of horizontal sections for an 8‐week‐old Lpar1‐EGFP mouse brain, imaged by light‐sheet fluorescence microscopy. Sections were taken at 200 µm intervals. White arrows indicate the Lpar1‐EGFP+ SP layer.

Although previous studies attempted to account for these volume changes (Torres‐Reveron and Friedlander 2007; Woo et al. 1991), their estimations were not specific to SP/L6b. To address this, we visualized the entire distribution of SpNs in the whole neocortex using the CUBIC tissue‐clearing method and employing LSFM at P0 and 8 weeks to quantify the SP/L6b volume (Matsumoto et al. 2019). We used the Lpar1‐EGFP transgenic mouse line (Hoerder‐Suabedissen and Molnar 2013; Walker et al. 2016) to define the SP, which consistently expresses GFP in the neurons located at SP/L6b and can be leveraged to distinguish the boundary between L6a and SP/L6b during postnatal development (Walker et al. 2016; Figure 11E). We defined the SP in P0 and L6b at 8 weeks based on the GFP signals and measured the areas of each slice to calculate the volume of the whole SP and L6b from z‐stack compilations (Figure 12A). The resulting SP volume was 0.57 ± 0.03 mm^3^ (*n* = 4), and the L6b volume was 4.22 ± 0.11 mm^3^ (*n* = 4), showing that the adult L6b exhibited a 7.4‐fold increase in volume, compared with that of the SP at P0 (Figure 12B).

**FIGURE 12:**
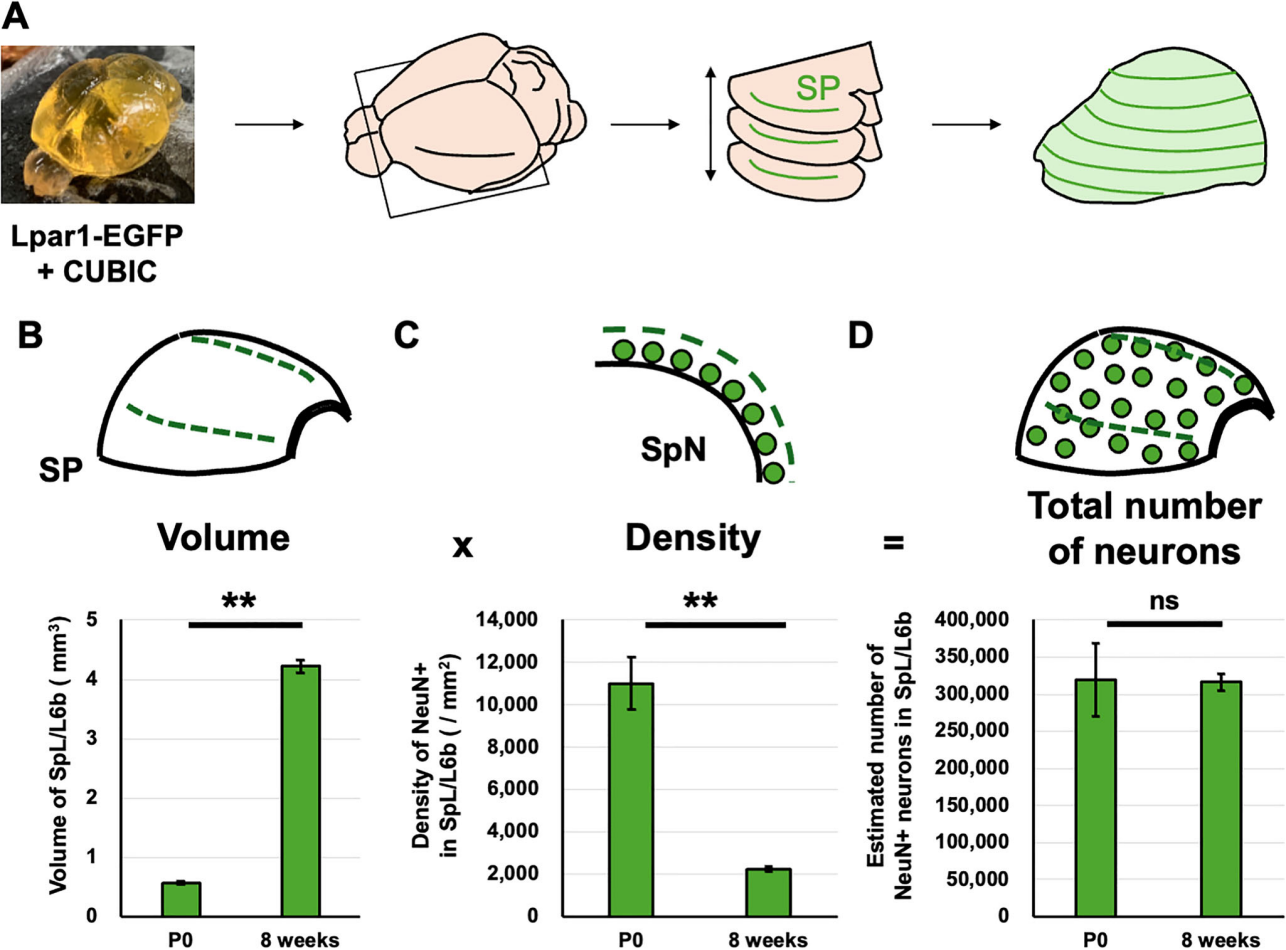
The total number of SpNs remains largely unchanged during postnatal development. (A) Schematic illustration of the method used to measure the volume of the SP layer and L6b across the entire neocortex. The black outlined square indicates the field of view for each image acquired by LSFM (left). Green lines mark the regions containing Lpar1‐EGFP+ cells in each acquired horizontal section (middle). The reconstructed 3D structure of the SP/L6b layer is shown on the right. (B) 3D reconstruction of the SP layer. Comparison of the SP volume at P0 and the L6b volume at 8 weeks (*n* = 4). (C) Schematic diagram of SpNs within a cortical slice. NeuN+ cell densities in the SP at P0 and L6b at 8 weeks were quantified from six fields of view per animal (n = 4 animals). (D) Schematic diagram of the 3D reconstruction for the SpN distribution throughout the SP. Estimated total number of neurons in the SP at P0 and L6b at 8 weeks (*n* = 4). ^∗∗^ *p* < 0.01, not significant (ns); Welch's *t*‐test (unpaired two‐tailed).

Next, we estimated the total number of SpNs at P0 and L6b neurons at 8 weeks in the entire brain. Due to the limited spatial resolution of LSFM, directly counting the GFP+ SpNs was not feasible. Therefore, we estimated cell numbers by combining cell density and volume measurements.

Although the expression of pan‐neuronal markers, such as NeuN, can change during postnatal development, SpNs mature earlier than other cortical neurons. Previous work has also shown that NeuN expression remains stable in postnatal SpNs (Hoerder‐Suabedissen and Molnar 2013). Therefore, NeuN was used as a reliable pan‐neuronal marker for quantification. We calculated the densities of SpNs and L6b neurons based on NeuN+ cells at P0 (11,016.42 ±1232.68 cells/mm^2^, *n* = 4) and at 8 weeks (2256.68 ± 107.25 cells/mm^2^, *n* = 4; Figure 12C). By multiplying these neuronal densities by the respective SP and L6b volumes and dividing by the width of the z‐axis slide section (20 µm at P0 and 30 µm at 8 weeks), we estimated the total number of SpNs as 319,588 ± 49,263, and the number of L6b neurons as 316,720 ± 11,268 (Figure 12D). This indicates that the decrease in SpN number was only 0.9%, with a loss of 2868 cells. Additionally, to determine whether this estimation accurately reflects the actual number of SpNs/L6b neurons at each stage, we quantified E12wl L6b neurons across the entire neocortex using serial coronal sections (Figure 13A). The 3D reconstruction of these data revealed that E12wl L6b neurons are distributed throughout the deep region of the neocortex (Figure 13B). To further examine their spatial distribution, we quantified E12wl EdU+ L6b neurons across more than 200 serial sections and calculated the cell density along the anteroposterior axis. The density of E12wl EdU+ L6b neurons was relatively uniform in the anterior regions and showed a slight increase posteriorly (Figure 13C). To evaluate whether NeuN‐based quantification accurately reflects SpN numbers, we performed the same estimation using the density of EdU‐positive cells labeled at E12, which specifically mark early‐born SpNs. These estimates were then compared with direct counts obtained at 8 weeks of age, in which all EdU‐positive cells were exhaustively quantified. The number of E12‐labeled neurons obtained by direct whole‐neocortex counting was approximately 1.6 × 10^5^ cells, corresponding to roughly half of the total estimated L6b population (3.2 × 10^5^ cells) (Figure 13D). This proportion was lower than the value predicted from the E12wl EdU+/NeuN+ ratio measured in the somatosensory cortex (64%), suggesting that birthdate‐defined L6b populations may not be uniformly distributed across the neocortex.

**FIGURE 13:**
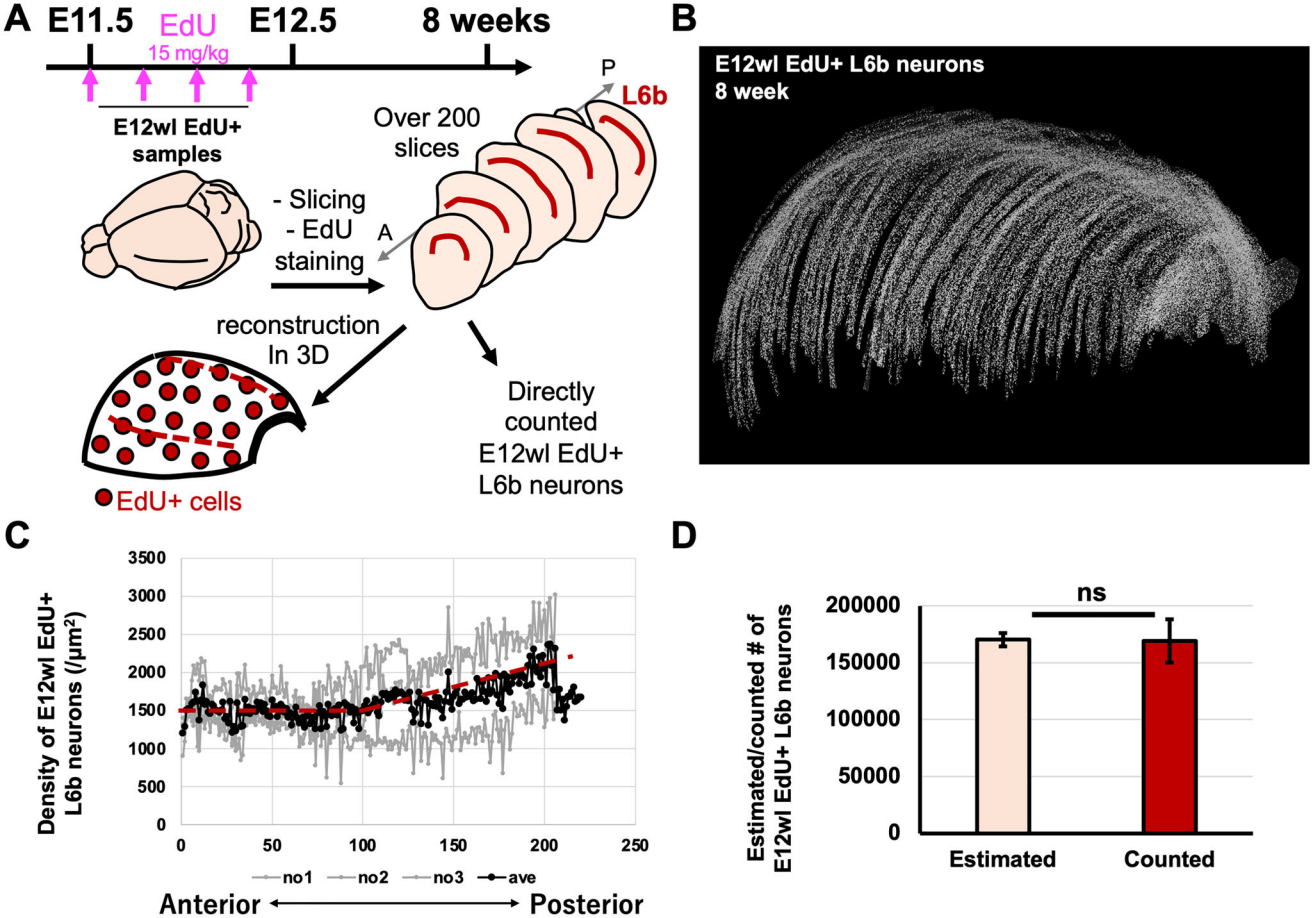
The distribution of E12wl EdU+ L6b neurons in the entire neocortex. (A) Schematic illustration of the method used to evaluate the estimation method, compared with actual cell counting. E12wl EdU‐injected mouse brains were sliced into over 200 serial coronal sections. EdU+ L6b neurons were counted, and L6b regions were reconstructed in 3D for visualization. Anterior: A, Posterior: P. (B) The 3D‐reconstructed half‐hemisphere of the sequential images for E12wl EdU+ L6b neurons at 8 weeks. (C) Density profile of E12wl EdU+ neurons in L6b along the anteroposterior (AP) axis. Gray lines represent individual data, and the black line shows the mean (*n* = 3 brains). Not significant (ns); Welch's *t*‐test (unpaired two‐tailed) (D) Comparison between the directly counted number of E12wl EdU+ L6b neurons across the whole neocortex and the estimated number predicted from somatosensory cortex‐based sampling.

Previous studies estimated the number of SpNs using the neocortical expansion rate during postnatal development. To determine whether the neocortex and SP/L6b expanded at different rates, we measured the volume of the neocortex using the same method at P0 and 8 weeks. The gray matter, defined as regions above WM and including SP/L6b (delineated by red lines in Figure 14A), increased from 11.29 ± 1.04 mm^3^ at P0 (*n* = 4) to 135.13 ± 4.08 mm^3^ at 8 weeks (*n* = 4, Figure 14B), consistent with volume measurements performed at respective stages by MRI (P0: 13.8 mm^3^, P80: 130.8 mm^3^; Zhang et al. 2005). Accordingly, the volume expansion rate of the neocortex was approximately 12.0‐fold from P0 to 8 weeks (Figure 14B). Considering that the expansion rate of SP/L6b was approximately 7.4‐fold, these results indicate that the neocortex expands more than SP/L6b. Moreover, the results imply that previous studies used a higher SP expansion rate than the actual one and overestimated the number of SP neurons in the adult. Therefore, the decrease in SpN density is primarily attributable to the expansion of the neocortex during postnatal development rather than to significant cell loss (Figure 14C).

**FIGURE 14:**
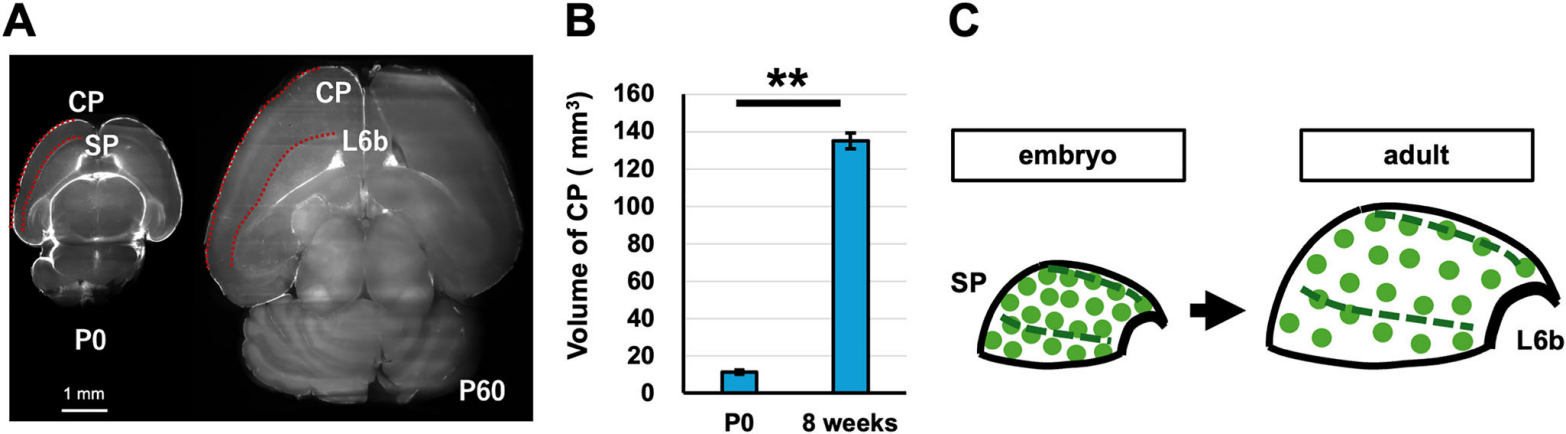
Expansion of cortical plate in postnatal development. (A) Horizontal sections of the whole brain for the Lpar1‐EGFP mouse at P0 (left) and 8 weeks (right). The red dashed line indicates the outline of the neocortex and the boundary between the neocortex and the SP layer/L6b. Scale bar = 1 mm. (B) Comparison of the CP volume at P0 and 8 weeks (*n* = 4). (C) Schematic illustration of the postnatal expansion of the SP/L6b region. Green dots represent SpNs and L6b neurons. ^∗∗^ *p* < 0.01, not significant (ns); Welch's *t*‐test (unpaired two‐tailed).

### 4.6 Layer‐Specific Estimation of Neuron Numbers Efficiently Reflects a Decrease

We next evaluated whether this estimation method accurately reflects the actual reduction in neuron numbers when neurons are eliminated. We focused on another early‐born neuron population, CR cells, which are known to decrease through apoptosis during postnatal development (Anstötz et al. 2014; Ledonne et al. 2016; Zecevic and Rakic 2001). The number of EdU+ cells in L1 was reduced at P14 (Figure 2G) and 8 weeks (Figure 15A). Using the same tissue‐clearing method of the whole brain, we measured the volume of the MZ/L1 region (Figure 15B). The MZ/L1 volume was 1.96 ± 0.24 mm^3^ (*n* = 4) at P0 and 13.50 ± 1.75 mm^3^ (*n* = 4) at 8 weeks (Figure 15C), indicating approximately a 7‐fold increase. We next analyzed the density of NeuN+ in MZ/L1 at P0 and 8 weeks. The density was 2745.60 cells/mm^2^ at P0 and 242.83 cells/mm^2^ at 8 weeks, showing an 11.3‐fold decrease (Figure 15D). We then multiplied the volume and density to estimate the number of MZ/L1 neurons, observing a decline from 269,411 at P0 to 106,153 at 8 weeks (Figure 15E), a decrease of approximately 61%. Considering that neurons were defined based on the NeuN expression, the number of neurons in MZ/L1 may include L1 interneurons (Schuman et al. 2019). However, because not only CR cells but also interneurons decrease during postnatal development, the 61% decrease in MZ/L1 neurons was reasonable. These results indicate that our estimation method successfully captures the actual reduction in the total neuron numbers. Furthermore, this approach enables the analysis of neuron number dynamics without the confounding effects of neocortical expansion during postnatal development.

**FIGURE 15:**
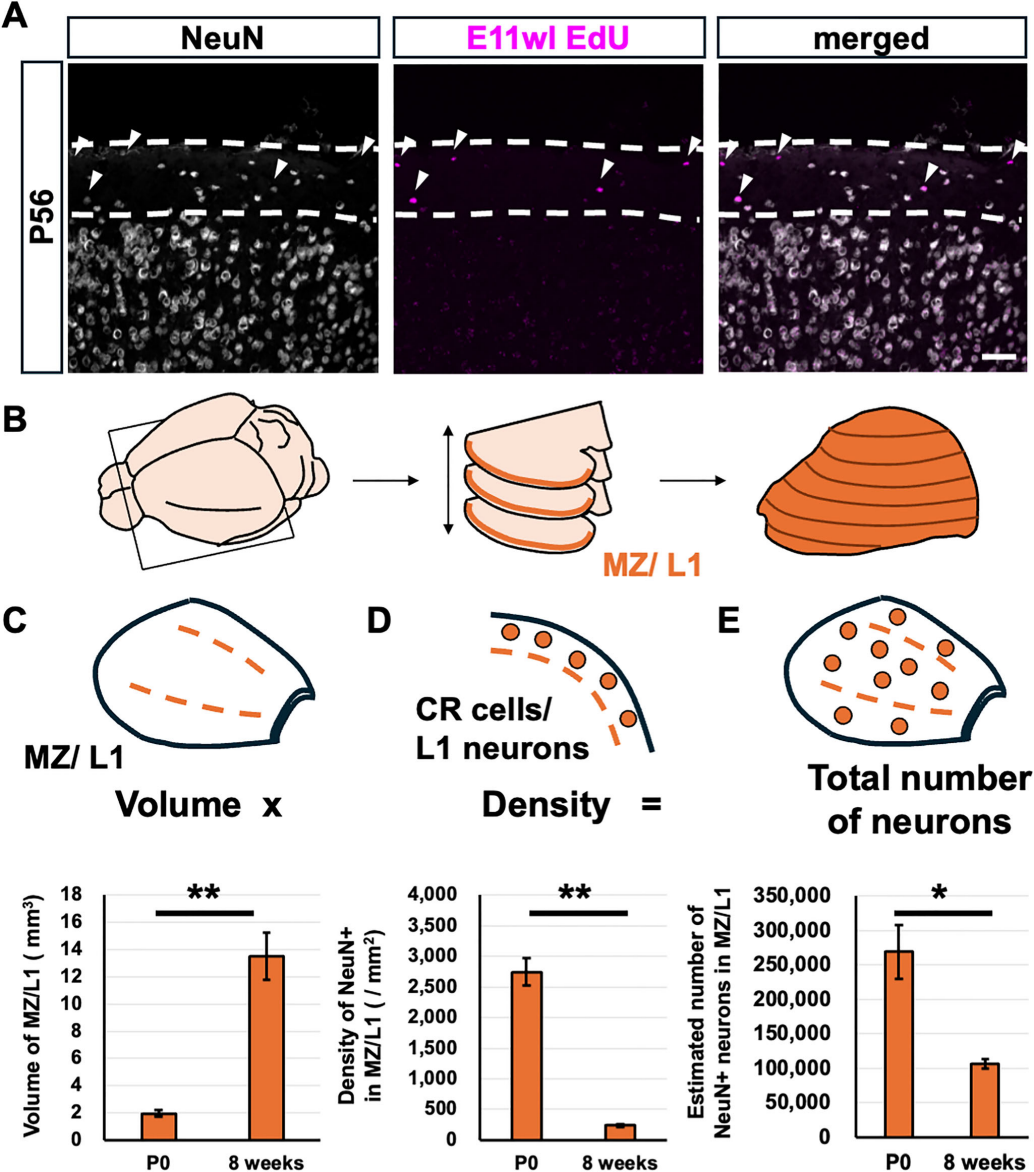
The decrease in CR cells is detected using the 3D estimation method of the cell number. (A) Immunohistochemical images showing the distribution of E11 EdU+ cells in the adult L1. NeuN (white) and EdU (red) staining at 8 weeks. White arrowheads show EdU+ cells. White dashed lines delineate the L1 boundary. Scale bar = 50 µm. (B) Schematic illustration of the method used to measure the MZ/L1 volume across the entire neocortex. The black outlined square indicates the field of view for each image acquired by LSFM (left). Orange bands indicate the MZ/L1 regions in each horizontal section (middle). The reconstructed 3D structure of the entire MZ/L1 layer is shown on the right. (C) Comparison of the MZ volume at P0 and the L1 volume at 8 weeks (*n* = 4). (D) NeuN+ cell densities in the MZ at P0 and L1 at 8 weeks (/mm^2^; *n* = 6 fields of view, 3 animals). (E) Estimated total number of neurons located in the MZ at P0 and L1 at 8 weeks (*n* = 4). ∗*p* < 0.05, ∗∗*p* < 0.01; Welch's *t*‐test (unpaired two‐tailed).

## 5 Discussion

SpNs are essential for embryonic neocortical development, yet they have long been thought to disappear after birth in the mammalian neocortex. In this study, we demonstrated that a significant number of early‐born SpNs labeled by EdU survive through postnatal development, and that the decrease in SpN density is primarily due to the expansion of the neocortex rather than cell death during postnatal development. While some studies have suggested the connection between SP and L6b neurons in rodents (deFazio et al. 1987; Pedraza et al. 2014), definitive evidence was lacking to prove this. By employing long‐term birthdate labeling and tissue clearing, we resolved this issue. Our results reveal that SpNs, defined as early‐born neurons (E10.5–E12.5), constitute a large proportion of L6b neurons in the adult neocortex, with surprisingly little change in the total number of SP/L6b neurons between P0 and 8 weeks. Additionally, L6b neurons in adulthood expressed canonical SpN markers, further supporting the conclusion that SpNs persist in adult L6b, beyond their role in embryonic neocortical development.

We developed an efficient estimation method to quantify layer‐specific neurons, including SpNs/L6b and CR/L1 neurons, and confirmed that this method successfully reflects the actual number of E12wl EdU+ SpNs. Still, there were two limitations to this method. First, CUBIC treatment can cause tissue expansion or deformation, which may affect volumetric measurements of the SP/L6b, CP, and MZ/L1. To address this issue, we compared our CUBIC‐based volume estimates with the publicly available MRI‐based measurements and found that the overall difference was relatively small. However, this comparison does not fully correct for potential tissue deformation caused by CUBIC treatment. Second, the directly counted number of E12wl EdU+ L6b neurons accounted for approximately half of the total estimated adult L6b neuron population, whereas the E12wl EdU+ ratio measured in the somatosensory cortex was approximately two‐fold higher than the E11wl ratio. This discrepancy suggests that birthdate‐defined SpN/L6b populations may not be uniformly distributed across the entire neocortex. Consistent with this possibility, our analysis in Figure 13C showed that E12wl EdU+ L6b neurons tended to be more densely distributed in posterior cortical regions than in anterior regions. To address this, we minimized regional bias by collecting images from multiple areas and averaging the results. For future studies that quantify the total number and subtype composition of SpNs—defined by molecular markers or birthdate—combining whole‐tissue clearing with genetic labeling is expected to allow direct enumeration of all SpNs. Nevertheless, our long‐term birthdate labeling proved to be markedly more efficient than a single analog injection, increasing the efficiency rate by approximately 6‐fold. Notably, in E12wl, despite four EdU injections within 24 h (equivalent to a 6‐h labeling window), the EdU^+^ percentage was higher than expected. Considering that approximately 40% of SpNs are derived from intermediate progenitor cells (Vasistha et al. 2015), some SpNs originating from intermediate progenitor cells may undergo division after EdU incorporation.

While our methods elucidated the distribution of early‐born neurons in L6b, this birthdate analysis could not identify the precise origin of the SpNs. While most SpNs are thought to arise from the ventricular zone, some SpNs are reported to migrate from other regions, including the rostromedial telencephalic wall (RMTW; Pedraza et al. 2014) and dorsomedial side of the cortical hem (Saito et al. 2019). RMTW neurons are generated around E11, then migrate tangentially to the cortical plate and sparsely distribute throughout the SP (Pedraza et al. 2014). Pedraza et al. (2014) proposed that the SpN population generated from E10 to E11 is mainly derived from the RMTW, whereas other SpNs generated at E12 are derived from the ventricular zone. Combining these studies with our results, E11wl SpNs may be derived from the RMTW. Furthermore, differences in molecular expression between E11wl and E12wl SpNs (more E12wl SpNs expressed NR4A2) suggest distinct subtypes based on birthdate and origin. Future studies focusing on these molecular and developmental differences may help classify early‐born SpNs into several subtypes and identify their heterogeneous molecular character.

In this study, we revealed that SpNs survive as L6b neurons in the adult neocortex; however, their specific contributions to the adult neocortical circuits and mechanism underlying their functional transition from embryonic SpNs to adult L6b neurons remain unclear. Previous studies have demonstrated that L6b neurons are active and contribute to functional neuronal circuits with specific modalities, including visual and auditory processing (Chang et al. 2024; Yoneda et al. 2023). Yoneda et al. (2023) identified L6b neurons based on CTGF expression, recorded their neuronal activity, and demonstrated their plasticity within functional circuits of the adult primary visual cortex. In our study, we revealed that EdU+ L6b neurons also express CTGF (Figure 7A–C), indicating that surviving SpNs in the adult L6b participate in mature functional circuits in the adult neocortex. To gain deeper insight into their role, future studies should investigate the molecular and electrophysiological mechanisms that drive the postnatal maturation of SpNs into functional L6b neurons.

We demonstrated that many early‐born SpNs survive in the adult L6b, with minimal differences in their number between the SP at P0 and L6b at 8 weeks in the mouse neocortex. However, developmental changes of SpNs in other species, especially in primates, including humans, are considered significant. A study focusing on cats revealed a ∼90% reduction of the SpNs during neocortical development, primarily thought to be eliminated by cell death (Luskin and Shatz 1985). Studies have also suggested that in the human neocortex, drastic changes occur in the SP with expansion and a decrease in depth during embryonic neurogenesis (Adorjan et al. 2019; Huang et al. 2009; Judaš et al. 2013; Kanold 2009; Kostović et al. 2011; Kostović et al. 2019; Kostovic 2020). These primate‐specific changes in the SP may contribute to the formation of a more complicated and diverse neuronal circuit that enables advanced information processing, although the detailed mechanism of SpN death remains unclear in these species. Therefore, it is likely that species‐specific genetic networks regulate differences in SpNs in neocortical development.

In conclusion, our study revealed that SpNs, which play crucial roles during embryonic development, survive and contribute to the adult neuronal circuits in mice. Further experiments are needed to elucidate the functional significance and activities of surviving SpNs as L6b neurons in the adult neocortex.

## Author Contributions

Y.S., K.M‐I., and C.O‐M. designed the study; Y.S. and K.M‐I. performed the research; Y.S., K.M‐I., and C.O‐M. analyzed data; Y.S., K.M‐I., C.H., and C.O‐M. wrote the paper; C.H. provided advice and expertise and contributed to the manuscript; C.O‐M. provided financial and administrative support, supervised the research, and contributed to the manuscript.

## Funding

This work was supported by the Japan Agency for Medical Research and Development (AMED; Grant Number 23gm1310012 to C.O‐M.) and JSPS KAKENHI grants (20H03270 to C.O‐M., 25KJ2147 to Y.S.). FY2021 Research Grant from the Yamada Science Foundation, the KOSE Cosmetology Research Foundation, the Mitsubishi Foundation, and the Astellas Foundation for Research on Metabolic Disorders to C.O‐M.

## Conflicts of Interest

The authors declare no conflicts of interest.

## Data Availability

The data that support the findings of this study are available from the corresponding author upon reasonable request.
